# Pediatric neurobrucellosis associated with hydrocephalus

**DOI:** 10.4103/1817-1745.76115

**Published:** 2010

**Authors:** Murat Altas, Omer Evirgen, Vefik Arica, Murat Tutanc

**Affiliations:** Departments of Neurosurgery, Mustafa Kemal University, Hatay, Turkey; Infectious Diseases, Mustafa Kemal University, Hatay, Turkey; Pediatric, Mustafa Kemal University, Hatay, Turkey; Family Medicine Tayfur Ata Sökmen Medical Faculty, Mustafa Kemal University, Hatay, Turkey

**Keywords:** Brucellosis, hydrocephaly, neurobrucellosis

## Abstract

Brucellosis is an infectious disease, frequently encountered in developing countries. It may involve multiple organ systems of the human body. However, neurobrucellosis is a rare complication of brucellosis. The most frequent events of cranial involvement are meningitis and meningoencephalitis. In the present case, a 10-year-old girl was referred to our clinic with fever, headache, nausea, and vomiting. The patient’s blood and cerebrospinal fluid cultures were found positive for brucellosis. Communicating hydrocephalus was also present in the cranial computed tomography as a complication of neurobrucellosis. The patient was successfully treated by external ventricular drainage and triple antibiotic therapy. There was no need to insert a ventriculo-peritoneal shunt.

## Introduction

Brucellosis is a chronic granulomatous infection. *Brucella*, the bacteria causing brucellosis, spreads from animals to people, directly or indirectly, often via consumption of unpasteurized milk, cheese, and other dairy products made from infected animal products. This zoonotic disease may involve almost the entire organ systems of the human body. Primarily, it affects the reticuloendothelial system (RES) and the osteoarticular system. The condition may lead to clinic morbidity, as it becomes significant in the rural parts of developing countries where uncontrolled dairy products are widely consumed. The condition has a high prevalence particularly in countries at the Mediterranean basin and Middle East.[1] In brucellosis, the central nervous system (CNS) involvement is a rare event, and it may affect 5-7% of patients.[2,3] CNS involvement usually includes miscellaneous conditions such as meningitis, encephalitis, meningoencephalitis, meningovascular disease, brain abscess, and demyelination problems.[1] The diagnostic criteria in neurobrucellosis include unexplained neurological symptoms, the Wright agglutination test positivity over 1/160 for *Brucella*, increased protein and lymphocyte count, and a positive Wright agglutination test in cerebrospinal fluid (CSF) even with very low titers, and a dramatic response to antibiotic therapy.[4]

Clinical categorization of adult neurobrucellosis cannot be applied to children because neurobrucellosis usually affects the CNS and presents itself in an acute form in children. The period of symptoms may vary between 0.5 and 8 weeks.[2,5] In this report, neurobrucellosis with hydrocephalus has been discussed in a 10-year-old girl.

## Case Report

**Figure 1:**
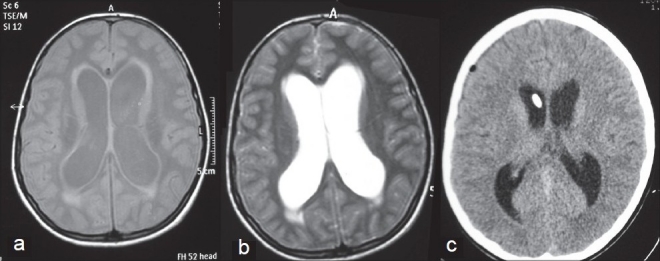
(a) T1W (flair) and (b) T2W brain magnetic resonance imaging (MRI) shows communicating hydrocephalus and periventricular edema. (c) Axial CT demonstrates the catheter for external ventricular drainage in the frontal horn of the right lateral ventricle, decreased ventricular size, and disappearance of periventricular edema

A 10-year old girl was referred to our clinic for complaints of fever, headache, nausea, vomiting, lethargy, and urinary incontinence. No specific feature was present in her medical history. The patient experienced a normal psychomotor development. Clinical examination revealed a temperature of 38.5°C. Lethargy, nuchal rigidity (++), positive Kernig’s and Brudzinski’s signs, isochoric pupils, direct and indirect light reflexes (+) were detected during neurological examination. Posture and walking were found unstable during the examination of the motor system. Romberg (++) and cerebellar tests were competent. No loss of strength in bilateral upper and lower extremities was determined. Deep tendon reflexes were hypoactive, and pathological reflexes were absent. Grade I papilloedema was observed on ophthalmological examination. Cranial computed tomography (CT) demonstrated a significant temporal horn enlargement and a communicating hydrocephalus causing dilation of the 3rd and lateral ventricles [Figure 1].

Hemoglobin was 9.8 mg/dl, erythrocyte sedimentation rate was 40 mm/hour, C-reactive protein was 24 mg/l (0–5 mg/l), and white blood cell count was 3.700/mm^3^ with a 70% of lymphocyte ratio. Blood glucose and liver function tests were normal. In lumbar puncture (LP), pressure in CSF was increased and a turbidity, pandy test (++) appearance turbidity, pandy test (++) appearance was observed. Leukocyte count was 455/mm^3^ with 36% of neutrophils and 64% of lymphocytes. Protein value of CSF was 148 mg/dl. Wright agglutination test was positive with a ratio of 1:1280 in blood. Gruber–Widal test was negative, and Wright and Rose Bengal test was positive. After the diagnosis of neurobrucellosis, an external ventricular drainage was performed and the catheter was removed, 10 days after the insertion.

Hydrocephalus did not relapse after the removal of catheter, and therefore there was no need to insert a ventriculoperitoneal (V–P) shunt during follow-up. Rifampicin 15 mg/kg/day, doxycycline 5 mg/kg/day, and gentamicin 5 mg/kg/day were given for 6 weeks as an antibiotic therapy. Dexamethasone was administered 12 mg/day in three doses for 7 days, then decreased gradually, and stopped. Repeated CSF cultures were negative. The ventricular catheter was removed after all the hydrocephalic findings disappeared on day 14. At the end of the 6-week therapy, blood and CSF cultures became negative. Patient was then discharged from the hospital after all of the laboratory tests became normal.

## Discussion

Neurobrucellosis is a rare complication of brucellosis, but the course of the condition is very severe. It is seldom seen in children. Symptoms may include fever, headache, vomiting, fatigue, depression, back pain, muscle tension and spasms. Additionally, findings of meningeal irritation, deep tendon reflexes areflexia or positive Babinski plus, hyperreflexia and other findings of systemic brucellosis can be observed. Sensorial or motor abnormalities at different degrees, cranial nerve retention, convulsions, cerebellar dysfunction, coma and brain abscesses may worsen the condition.[6,7] Adult neurobrucellosis may present itself in an acute or chronic form. It may affect the central or peripheral nervous systems or, sometimes, may affect them both. Peripheral nervous system involvement may be seen alone or together with the CNS retention in nearly 1/3 of adult cases.[2,8]

The principal clinic presentation of neurobrucellosis in children is displayed as an acute meningitis or meningoencephalitis. Usually, brucellosis may not be included in CSF assessment for differential diagnosis. Therefore, the diagnosis of the condition can be difficult. Regularly, CT is assessed as normal in radiological evaluation in children. Cerebral atrophy, thickening of optical nerves, dilation of the lateral ventricle or cerebellar abscess is scarcely seen.[9–12] In our case, cranial CT demonstrated a communicating hydrocephalus. In several studies, granulomatous inflammation of the meninges and brain parenchyma was reported in radiological evaluation of patients with brucellosis. The reason that caused communicating hydrocephalus was reported as granulomatous inflammation of arachnoid’s villus.[13] In a recent article published by Panagariya *et al*., it was reported that a similar condition known as a pseudo-tumor accompanied 4% of the patients with neurobrucellosis.[14] In neurobrucellosis, pathological modifications can always be present in the meninges. Such pathological changes are more significantly seen in basal regions and are characterized by thickening of meninges, which depends on connective cell proliferation or acute/chronic inflammatory cellular infiltration and diffused interaction.[13]

The literature of pediatric neurobrucellosis displays therapeutic regimes of various periods and management regarding the treatment of the condition. The mentioned regimes consist of two or three drug combinations. The recent recommendations announced for the treatment of acute brucellosis involves a-6-week course of the therapy and addition of an aminoglycoside to avoid relapses.[9,15] Steroids are administered to treat arachnoid and cranial nerve retentions, optic neuropathy, and papilloedema. The management of adult acute neurobrucellosis includes the administration of a triple combination of antibiotics, which are the newest consensus, similar to the treatment of pediatric neurobrucellosis. Aminoglycosides are administered for a period of 2–4 weeks, while tetracycline or cotrimoxazole and rifampicin combinations are administered for a period of 8–12 weeks. The reason is that two combined drugs may lead to relapses or failures.

The most ideal anti-brucellosis therapy in children is using aminoglycosides (gentamicin) which can be administered during the first 2–4 weeks. Additionally, rifampicin and doxycyline can be administered to children above 8 years of age. Cotrimoxazole should be administered to children less than 8 years of age, instead of doxycycline. Therapy must continue for at least 6 weeks, and may be continued further in chronic cases. Even after an appropriate therapy, symptoms may worsen due to extreme output of *Brucella* antigens and immune response. Steroids are indicated in such circumstances.[16] In a study carried out by Tanir *et al*., pediatric neurobrucellosis was diagnosed in 2 out of 90 patients with neurobrucellosis and they were treated by means of a triple antibiotic therapy. Two patients were referred to our clinic due to symptoms related with meningoencephalitis and cerebral edema was determined by cranial CT.[16] In another study carried out by Mantur *et al*., three pediatric brucellosis patients out of 93 were diagnosed with neurobrucellosis, while three of the patients presented chorea, peripheral neuritis, and meningitis. It was reported that Wright tests were positive for CSF, but the cultures remained negative. These patients were also treated by a combination of triple antibiotics.[17]

The most frequent complications of neurobrucellosis are meningitis and meningoencephalitis. Furthermore, a wide spectrum of complications such as cranial nerve retention, aneurysm, hydrocephalus, myelitis and parkinsonism can also arise with the condition.[18,19] In our case, we primarily preferred to apply a ventricular drainage because of the possibility of contaminating the patient’s abdomen via seeding, whereas we were aware that the hydrocephalus was a transient condition. Daily CSF drainage by ventricular catheter helped very much to track the responses to the treatment and provide regression in findings related to intracranial pressure increase. The catheter was removed when discoloration of the CSF and the absence of growth in the CSF culture occurred. No sign of hydrocephalus was encountered during the follow-up. Hence, we suggest that there is no need to rush to place a V–P shunt in hydrocephalus cases induced by neurobrucellosis. However, cases must be followed as usual. In our case, ventricular catheter, triple antibiotic management and steroid therapy were very successful in the treatment of neurobrucellosis.

To conclude, early diagnosis and appropriate treatment may provide a dramatic response in pediatric neurobrucellosis with a better prognosis than adults. In endemic regions, neurobrucellosis should be scrutinized in patients with hydrocephalus.
